# No benefit of a proximal stem centralizer in cementing of a femoral prosthesis in human cadavers: Measurement of intramedullary pressure, cement penetration, cement mantle thickness, and positioning of the stem

**DOI:** 10.3109/17453674.2011.566137

**Published:** 2011-07-08

**Authors:** Juozas Petruskevicius, Thomas Lind-Hansen, Ramune Aleksyniene, Jens R Nyengaard, Poul T Nielsen, Kjeld Søballe

**Affiliations:** ^1^Department of Orthopedic Surgery; ^2^Department of Nuclear Medicine, Aarhus University, Aalborg Hospital, Aalborg; ^3^Stereology and Electron Microscopy Laboratory, Aarhus University, Aarhus; ^4^Department of Orthopedics, Aarhus University Hospital, Aarhus, Denmark

## Abstract

**Background and purpose:**

A proximal stem centralizer may be beneficial regarding cementing pressures, cement penetration, and stem alignment. We measured these parameters when cementing a mat-surfaced femoral component with and without the use of a proximal stem centralizer.

**Material and methods:**

8 femoral prostheses with proximal centralizers and 8 femoral prostheses without proximal centralizers were cemented according to third-generation cementing technique in 8 pairs of embalmed cadaveric femora. We recorded intramedullary pressures (peak levels, the area under the pressure curves and mean pressure) with 6 pressure transducers during stem cementation. Computer tomographic scanning of specimens was performed to evaluate stem alignment after surgery. Thickness of the cement mantle, cement penetration, and stem centralization at the metaphyseal part of the femur were measured on cross sections using stereology.

**Results:**

There were no statistically significant differences in measured pressure and cement penetration values between the groups. There was similar cement distribution around the stems; however, in using a proximal centralizer, the cement mantle tended to be thinner laterally. Moreover, we found a larger variation in stem alignment on lateral projection in the proximal centralizer group.

**Interpretation:**

No benefits regarding intramedullary pressures and cement penetration were obtained from cementation of a straight stem with a proximal stem centralizer. However, there was an increased risk of inferior stem positioning in the reamed medullary cavity using the centralizing device.

An adequate cement mantle is important for long-term fixation of the cemented femoral component in hip arthroplasty (Malchau et al. 2002, Berry 2004). While the cause of aseptic loosening of femoral implants is multifactorial, central positioning of the stem in the medullary cavity is preferable regardless of implant geometry, implant surface finish, or implant design. The use of distal stem centralizers helps to control alignment of the stem, avoiding direct contact between the bone and the tip of the prosthesis (Egund et al. 1990, Berger et al. 1997). However, this device alone cannot prevent cement mantle deficiencies, especially in the proximal region of the femur (Berger et al. 1997, Crawford et al. 1999, Breusch et al. 2001).

Promising results using the proximal stem centralizer—regarding both prosthesis alignment and cement mantle thickness—have been reported in retrospective studies (Goldberg et al. 1998, Jarrett and Lachiewicz 2005). Experimental trials have also shown that a proximal centralizer can increase the intramedullary pressures in the proximal region of the femur, thereby enhancing the cement-bone interlock (Gozzard et al. 2003, 2005). Even so, no reports have been published on the relation between cementing pressures, cement penetration, cement mantle thickness, and the use of a proximal centralizer in a true-to-life study set-up. We compared these parameters during cementation of a Bi-Metric femoral prosthesis with and without a custom-made proximal stem centralizer.

## Materials and methods

We prepared 8 pairs of embalmed cadaveric femora, with a mean donor age of 77 (65–91 years). The cadavers had been preserved using a solution consisting of distilled water, glycerol, glutaraldehyde, glyoxal, 96% alcohol, and formaldehyde at the Institute of Anatomy, University of Aarhus. Before the experiments, the soft tissues were removed from the femora. Standard anteroposterior radiographs of known magnification were taken of all specimens before surgery to determine the correct size of the prosthesis. We determined the femoral bone type according to Dorr (1986); most femora were of type B (5 pairs), but 2 pairs were of type C and 1 pair was of type A. A straight, grit-blasted femoral stem of titanium alloy (Bi-Metric; Biomet) was used for cementation. Most stems were of size 9 (5 pairs), while size 7 was used in 2 pairs and size 11 in 1 pair. 4 of the 8 left femora were randomly allocated to the proximal centralizer group and the other 4 to the control group, providing an equal number of right and left femora in both groups.

### Design of the proximal centralizer

**Figure 1.**
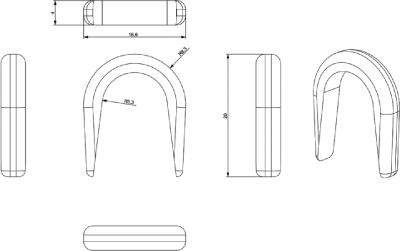
Design of the custom-made proximal stem centralizer.

The proximal centralizer (Figure 1) was custom designed to fit the medial part of the stem just below the meeting point between the neck and the body of the prosthesis. The rationale of the design was to prevent stem contact with inner bone contour, and to ensure sufficient thickness of cement medially. In addition, we expected that occlusion of the femoral canal medially would cause an increase in cementing pressures and deeper penetration of cement into the proximomedial region of the femur.

**Figure 2.**
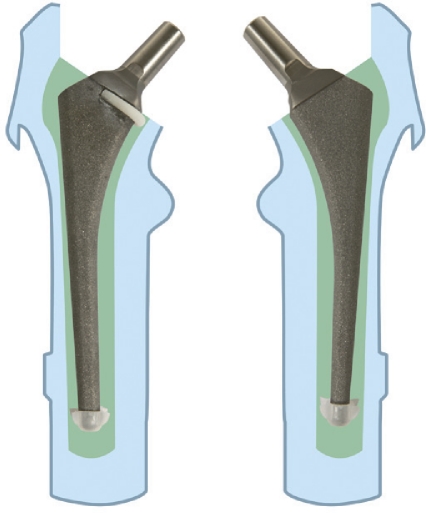
Femoral components. A proximal centralizer on the left stem (investigational group) and distal centralizers on both stems.

The centralizer was hemispherical, 2 mm thick at its rounded part, and became thinner (evenly) at both the anterior side and the posterior side of the stem. The branches reached approximately two-thirds of the width of the stem. The centralizer, 4 mm in height, was made of polyethylene powder from a 3-D drawing using a Rapid Manufacturing Machine (Danish Technological Institute, Aarhus). The polyethylene powder was placed in a special chamber where a laser beam was used to weld the substance into a 3-D form. 3 different sizes in terms of inner diameter were made to fit the commonly used stem sizes 7, 9, and 11. The centralizers were glued onto the prostheses before the cementation (Figure 2). All prostheses were equipped with a distal centralizer (Biomet) on the tip of the stem.

### Preparation of the femoral canal, and cementation technique

The femora were fixed in a vertical position with pipe-clamps. The femoral canal was opened with a rounded osteotome in the fossa piriformis. Straight medullary reamers were used to ream the canal until firm contact with the inner cortex was achieved. Then the femoral neck osteotomy was done with an oscillating saw 1.5 cm above the lesser trochanter, using the femoral resection guide in conjunction with the intramedullary reamer. The proximal part of the femur was broached with standard, sequentially larger, Biomet broaches, until either stability was achieved or the stem size selected during preoperative planning had been reached. The last broach used was 4 mm oversized compared with the stem. This technique should provide a 2-mm cement mantle if the stem is placed centrally in the reamed medullary cavity. The femoral canal was occluded with a polyethylene plug (Allen medullary cement plug; Zimmer) according to the size of the medullary cavity, 2 cm below the expected level of the tip of the prosthesis. The canals were cleaned using a high-pressure pulsatile lavage (OptiLavage; Biomet) with 1 L of warm saline. All the cement mixing and cementation was performed by one person (JP) to reduce variability. Operating room temperature was maintained at 20°C. 80 g of Refobacin high-viscosity bone cement was vacuum-mixed and injected with a cement gun (Optivac; Biomet) in a retrograde manner, 2 min after the start of mixing. The time when monomer and polymer first came into contact was considered to be the start of cement mixing. The cement was stored at room temperature. It was pressurized for 1 min by a silicone femoral pressurizer adapted to the delivery syringe. The stems were inserted manually in one continuous movement using the inserter handle, 4 min after the start of mixing. The components with the proximal centralizer were pressed down until the centralizer was below the bone-cutting level medially. The surgeon attempted to align all prostheses in a neutral position. The duration of insertion was about 30 sec. The medial part of the femoral neck was occluded with the thumb during the insertion of the stem; pressure was maintained on the stem through the inserter handle until the cement had polymerized.

### Recording of intramedullary pressure

We used a previously described method for recording intramedullary pressure (Reading et al. 2000).

**Figure 3.**
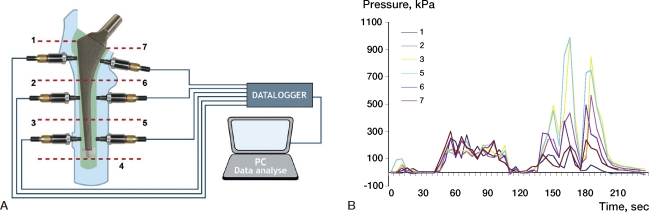
A. Schematic illustration of the positions of the pressure transducers and pressure recorder. B. A typical pressure profile during cementation is shown. Small elevations in pressure at the start of the curves correspond to cement application and pressurization whereas large peaks are related to stem insertion. 0 on the x-axis corresponds to cement application into the canal.

6 holes at the center of each Gruen zone (except zone 4) were drilled with a 3.8-mm drill, and the pressure transducers (Kulite Semiconductor Products, Leonia, NJ) were firmly threaded into the holes. The transducer tips had a piezoresistive sensor behind a metal diaphragm, which was flush with the cancellous bone contour (Figure 3). The pressure transducers were connected to a 6-channel data logger (Almemo 8990-8, Holzkirchen, Germany) and to a personal computer, which allowed simultaneous recordings. The transducers were calibrated to measure pressures from 0 to 1,700 kPa, and zero calibration was done after the pulsatile lavage before cementation. The intramedullary pressures were continuously recorded, beginning approximately 2 min before the injection of cement into the canal and ending when the intramedullary pressure returned to 0 (approximately 2 min after insertion). The measurements were performed at 5-second intervals. The sensitivity of the whole system (transducers, chart recorder, and PC software) was 0.34 kPa. We analyzed 3 pressure values: the peak pressure, the area under the curve (AUC), and the mean pressure (AUC/duration of stem insertion in seconds) during the stem insertion phase. The pressures during cement injection and during cement pressurization prior to stem insertion were not subjects of interest in this trial.

The pressure values were calculated and then compared between and within the groups at the medial and lateral, and also the proximal and distal Gruen zones. The medial and lateral zones at the same level were grouped together, allowing comparison of results at 3 levels: the proximal region (Gruen zones 1 and 7), the middle region (zones 2 and 6), and the distal region (zones 3 and 5).

### CT-scanning and sectioning of specimens

**Figure 4.**
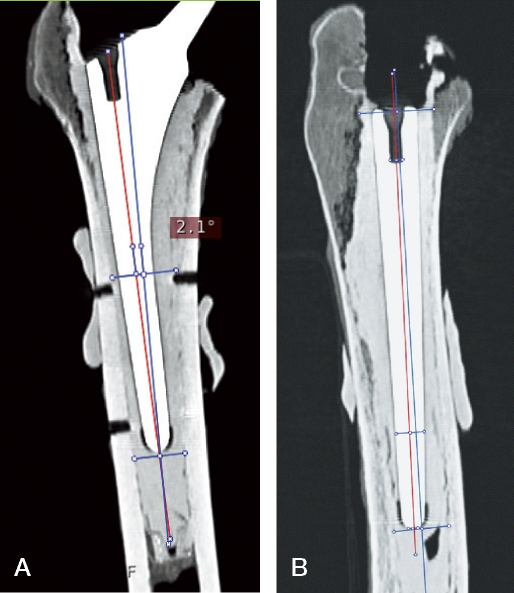
The axes of implant and femoral canal on both coronal (A) and lateral projections (B). Red line: axis of the femoral stem; blue line: femoral axis.

Each femur was scanned in a CT-scanner after the surgery. 3-D CT analysis was carried out using the medical data imaging software EasyViz (Medical Insight A/S, Denmark), which allowed evaluation of stem alignment in relation to the reamed medullary canal on the coronal and lateral projections. The femoral axis on the coronal plan was defined as a line connecting 2 middle points of the medullar cavity—one point just below the tip of the prosthesis and the second point at the level of the center of Gruen zone 2 (Figure 4). The femoral axis on the lateral projection was defined similarly, i.e., the middle point of the femoral cavity at the tip of the prosthesis was connected to the middle point at the level of the opening of the femoral canal proximally. The implant axis was drawn between the tip of the prosthesis and the middle of the “insertion” hole, defined at the proximal part of the stem on both projections. The angle between these 2 lines was measured with a digital angle ruler, and alignment was defined in degrees. The minimum possible angle that could be measured was 0.1°. The alignment on both projections was measured by the same investigator, twice within 1 week, on the same CT images. The reproducibility of measurement technique (intraobserver variation) was 1° (SD of mean difference between 2 measurements).

After CT scans, the proximal part of each femur was sectioned transversely into 9 samples using a high-precision diamond cutting machine. The first cut was performed perpendicular to the femoral axis at the cut edge of the calcar and subsequent cuts were done at 4-mm intervals. From the 16 femora, 144 samples with a thickness of 4 mm were produced for stereological analysis.

### Stereological analysis

The upper side of each cross section was placed under a macroscope connected to the computer. Length measurements were made guided by stereological sampling principles (Baas 2008) and software (NewCAST; Visiopharm). We defined the prosthesis line connecting the most medial and the most lateral point of the stem on each cross section. The samples were then systematically randomly orientated relative to the geometrical x-axis to avoid bias of the measuring areas. This was achieved by selecting a random number (1^st^ RN) from 0 to 180°, which defined the angle of the prosthesis line to the x-axis for the first cross section. The next cross section was randomly rotated (clockwise for right femora and anticlockwise for left femora) by adding 30° to the 1^st^ RN. The following cross sections from the same specimen were then consequentially and systematically rotated according to the equation: ∠⃒_n_ = 1^st^ RN + (n – 1) × 30°,

where ∠⃒_n_ is an angle of the prosthesis line to the x-axis measured in degrees, n is a cross section number, and 1^st^ RN is a random number in degrees defining the position of the first cross section relative to x-axis.

**Figure 5.**
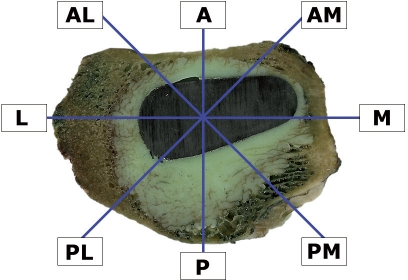
The nucleator with 8 intercepts defining 8 regions of interest (ROIs) on the cross section of the left femur. AL: anterior-lateral; A: anterior; AM: anterior-medial; M: medial; PM: posterior-medial; P: posterior; PL: posterior-lateral; L: lateral.

The prosthesis area, the inner contour of cancellous bone, and the outer contour of cement mass were determined for each sample. A 2-D nucleator (Gundersen. 1988) was used to determine the regions of interest (ROIs), because of the non-circular geometry of both cement and cancellous bone contours. The middle point of the nucleator with 8 intercepts radiating 45° relative to each other was approximated to the center of the prosthesis at each section (Figure 5). The touch point between the intercept and the contour of the prosthesis was marked, and the distances were measured from these points perpendicular to the contour of the prosthesis. 2 intervals were measured from each of the 8 points: the distance between the prosthesis and inner cancellous bone (W_cp_) and the distance between the prosthesis and outer cement contour (W_cw_). These two distances represented the width of pure cement mantle and of the whole cement mantle. The depth of cement penetration (P_d_) could be derived from these measurements: P_d_ = W_cw_ – W_cp_. All distances were measured in μm. The reproducibility was calculated as the coefficient of variation (CV) from the double measurements of each interval (Nilas et al. 1988). The coefficients of variation for W_cp_ and W_cw_ were 0.93% and 0.54%, respectively.

### Measurement of centralization of the femoral component

Centralization of the stem (ΔCp) was defined (in μm) by calculating the difference between the largest (Max_cp_) and the smallest (Min_cp_) thickness of the pure cement mantle: ΔCp = Max_cp_ – Min_cp_. If the thickness of the pure cement mantle is the same all the way around the prosthesis, the difference (ΔCp) will be 0, indicating perfect centralization. In contrast, a large difference will signify poor centralization. The mean value (from 9 cross sections per specimen) of the pure cement mantle at each ROI was calculated according to the stereological method described above. 2 regions with the thickest and the thinnest pure mantle were identified, and the difference between these 2 regions was derived.

### Statistics

The data were analyzed using Stata 9.0 (StataCorp, TX) and SigmaStat 2.0 (Jandel Corp., CA). The differences of pressure, cement penetration, and cement mantle measurements between the pairs are presented as mean ± SD and were analyzed using a multivariate analysis of variance (Hotelling T^2^; H0 difference between the pairs = 0) tests. Data were examined for both normal distribution and equal variances for both groups before statistical analysis. For the heterogeneously distributed data that were observed for stem alignment and stem centralization, Wilcoxon signed rank test was used. Variation in data of stem alignment and centralization was evaluated using Pearson's correlation test. The level of significance was set to < 0.05. The study was designed to reveal a difference in peak pressure of 120 kPa between the paired groups. We needed to perform at least 8 experiments in each group to show this difference, when SD of the difference was 100 kPa, type-I error was 0.05, and type-II error was 0.20.

## Results

Cementation of stems with proximal centralizer took approximately 10 seconds longer than in the control group, even though we attempted to complete the stem insertion in 30 sec. Pressure recordings took about 10 seconds longer to return to 0 in the proximal centralizer group than in the control group (mean 90 (SD 18) vs. 80 (23) seconds, measured from the beginning of stem insertion).

### Peak pressure

**Figure 6.**
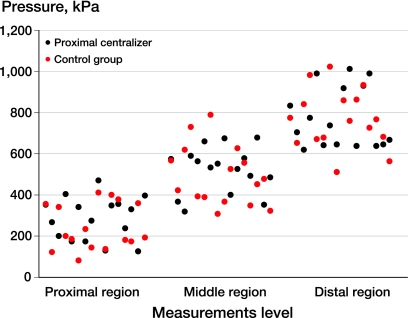
Peak pressure recordings during stem insertion. Measurements at the medial and the lateral zones belong to the same region.

Peak pressures were similar between the groups in all 3 regions (p = 0.8) (Figure 6 and Table 1). We found a clear trend of higher pressures distally for all 3 pressure parameters recorded (peak pressure, AUC, and mean pressure) in both groups (p = 0.003) (Figure 6). No statistically significant differences between the medial and the lateral Gruen zones at the same level were observed (p = 0.07 for control and p = 0.2 for proximal centralizer).

**Table 1.** Peak pressures at the proximal, middle, and distal regions attained during stem insertion

Region	Mean peak pressure (SD) and [95% CI], kPa		
	Proximal centralizer	Control group	Difference
Proximal	285 (104) [230−341]	243 (110) [184−302]	42 (134) [–29 to 114]
Middle	521 (113) [461−581]	491 (145) [414−569]	30 (194) [–74 to 133]
Distal	770 (148) [692−849]	765 (143) [689−841]	5 (185) [–94 to 104]

### AUC and mean pressure

Higher AUC values were measured in the proximal centralizer group than in the control group in all 3 regions, whereas the mean pressures were higher in the control group. These differences were not statistically significant (p = 0.8 for both AUC and mean pressure).

### Cement penetration

**Figure 7.**
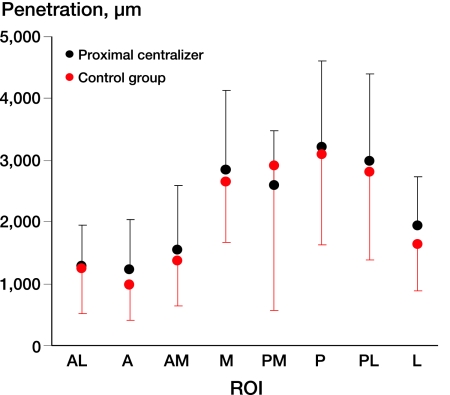
Mean cement penetration at 8 ROIs. The error bars represent SD. For abbreviations, see Figure 5.

No statistically significant differences regarding cement penetration were observed between the groups (p = 0.6). Deeper cement penetration was seen in the M, PM, P, and PL regions compared with the other 4 ROIs in both groups, but these changes were not statistically significant (p = 0.5 for changes between the regions, p = 0.9 for equivalent curves) (Figure 7).

### Thickness of the cement mantle

**Figure 8.**
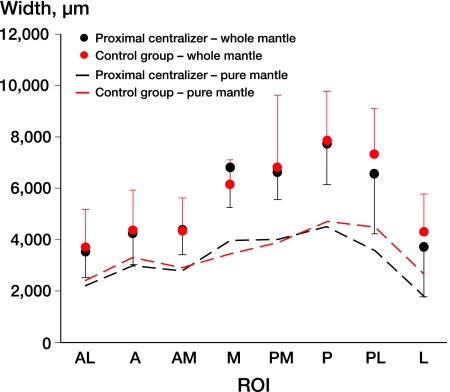
The thickness of the whole cement mantle and of the pure cement mantle in both groups. The error bars represent SD.

The distribution of whole and pure cement mantle around the stem was similar in both groups. A tendency of thicker cement mantles was noted at the M, PM, P, and PL regions compared with the other 4 regions; (p = 0.08 for whole mantle, p = 0.1 for pure mantle) (Figure 8). We found that both mantles were thicker in the medial region in the proximal centralizer group, while in the other regions (especially the posterior-lateral and lateral regions) thicker cement was observed in the control group. However, the statistical test of parallel curves did not reveal any significant differences between the groups in any of the regions (p = 0.9 for whole mantle, p = 0.5 for pure mantle). There were no statistically significant differences between the 8 ROIs within the groups (p = 0.9 for whole mantle, p = 0.7 pure mantle).

### Alignment of the femoral stem

**Figure 9.**
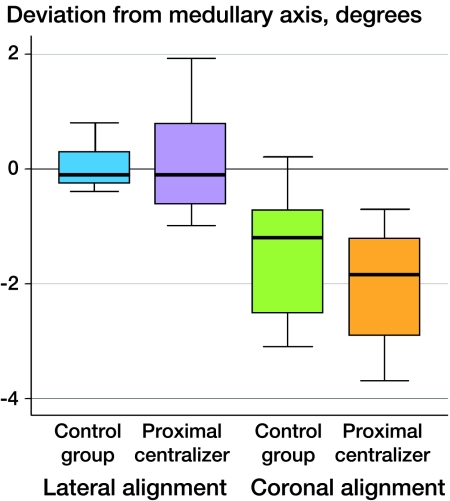
Data regarding stem alignment on coronal and lateral projections in both groups. The wiskers represents the lower and the upper adjacent value. Lower adjacent value = is the smallest value above the lower inner fence which is calculated by the formula: 25th percentile –1.5 x (75th percentile – 25th percentile).

Although the stems were slightly better aligned in the control group, deviations from both femoral axes were insignificant—both statistically and clinically (Table 2 and Figure 9). The distribution of data for stem alignment on lateral projection was different between the groups; significantly larger variation was seen in the proximal centralizer group (r = –0.74, p = 0.04). No differences in variance were found for coronal alignment (r = 0.07, p = 0.86) (Figure 9).

**Table 2.** Median deviation from the femoral axis (interquartile range), in degrees **^a^**

Alignment	Proximal centralizer	Control group	p-value
Coronal alignment	–1.8° (–2.9 to –1.2)	–1.2° (–2.6 to –0.7)	0.3
Lateral alignment	–0.1° (–0.7 to 0.8)	–0.1° (–0.3 to 0.3)	∼1.0

**^a^** Minus value represents valgus on coronal projection and posterior tilt on lateral projection.

### Centralization of the femoral component

We did not find any significant difference between the groups regarding stem centralization (p = 0.9); however, a slightly larger degree of variation of data was seen in the proximal centralizer group but this was not statistically significant (r = –0.6, p = 0.1).

## Discussion

Despite the fact that there has been much research regarding cement penetration into cancellous bone, only a few studies have investigated the factors influencing cementation pressures and cement intrusion at the most proximal femoral region when cadaver femora are used (Song et al. 1994, McCaskie et al. 1997, Dozier et al. 2000, Reading et al. 2000). It has been suggested that a proximal stem centralizer could increase the cementing pressure, preventing cement outflow proximally.

We found smaller pressure increase and larger variation than expected, i.e. no statistically significant difference was found between the groups. Some brief pressure peaks may have been missed because the measurements were recorded at 5-second intervals. However, we do not consider that these missed values would have had any clinical significance. These momentary elevations in pressure would not necessary mean sufficient pressure maintenance during stem insertion, because prolonged pressure application is usually needed (> 5 seconds) to achieve optimal cement penetration (Halawa et al. 1978, Krause et al. 1982).

We obtained the typical intramedullary pressure recordings, with highest pressures during stem insertion. Despite the presence of a proximal centralizer, the greatest values were still achieved at the distal end of the femoral canal. This is in agreement with most other studies (Oh et al. 1978, Song et al. 1994, McCaskie et al. 1997, Yee et all. 1999, Reading et al. 2000, Churchill et al. 2001, Munro et al. 2007).

We could not, however, confirm the results published by Gozzard et al. (2003, 2005), who conducted 2 experimental studies showing increased pressure in proximal regions during cementation of the CPS-Plus stem with a proximal centralizer. The reason for this discrepancy might be explained by a difference in laboratory models. Gozzard et al. used a round proximal centralizer, which may have yielded a better occlusion of the femoral cavity than our centralizer, but greater attention should be paid to the femoral molds. The femoral canals were made of dental plaster, which lacks cancellous bone interstices; therefore, cementation pressures recorded in that study would not be representative of those achieved in normal bone anatomy. This limitations of in-vitro studies using plastic femora or artificial molds have been pointed out previously (McCaskie et al. 1997, Munro et al. 2007).

We did not find any significant difference in the pressure values at the medial and lateral sides measured at the same level in both groups. This indicates that the design of our proximal centralizer fails to enhance the cement flow at the calcar region, allowing cement to escape through the lateral-proximal opening.

It should also be mentioned that we found larger standard deviations of pressure values than published from studies where simulated femur canals were used and stem insertion was fully automated. Some sources of variation such as the operative technique, the anatomical variation in the femurs, and the size of the prosthesis could be partially controlled by the paired design of the study. As already reported by others (Churchill et al. 2001), varying rheological properties of the cement can have a great influence on the results. We did not know the exact viscosity of the cement at the time of stem insertion because we used the time after mixing as an indicator of cure stage. This is perhaps the most important limitation of our study. Even so, we believe that our results better represent the clinical situation, where conditions are similar to those in the operating theater.

The stereological technique we used for image analysis allowed design-based evaluation of both the depth of cement penetration and cement thickness. Only the proximal 5 cm of each femur was used, because most of the cancellous bone is preserved there. As mentioned previously, the depth of cement penetration and cement mantle thickness were similar in both groups. The average depth of penetration in both groups was less than 4 mm, which has been claimed to be the optimal depth for strength at the cement-bone interface (Askew et al. 1984). Previous investigators have required only 76 kPa to achieve this depth of penetration, while substantially higher pressure values recorded in our study could not yield the same penetration level. This can be explained by the use of high-viscosity cement in our trial and the differences in canellous bone porosity.

We found a tendency of thicker cement in the antero-medial and medial regions (ROI: AM and M) in the proximal centralizer group than in the control group. This indicates that the proximal centralizer works in accordance with its design—i.e. creating a thicker cement mantle medially, but at the expense of the lateral regions of the femur. It appears that the proximal centralizer pushes the stem more laterally, resulting in an asymmetrical cement mantle around the prosthesis. Both cement penetration and the cement mantle were thinnest anteriorly. This confirms the previously reported observations that it is difficult to achieve sufficient cement mantle in this region when a straight stem design is used (Crawford et al. 1999).

Stem centralization in the metaphyseal part of the femur was similar in the 2 groups. However, the larger variation in the proximal centralizer group highlights the lower degree of precision of stem placement in the reamed canal. The reason might be anatomical variation of the femoral neck, influencing the position of the stem when the centralizer makes contact with the inner contour of the bone. The possibility of stem manipulation was inhibited because of reduced space in the femoral canal. More valgus deviation in the AP plan, with greater variation in the stem positioning on the lateral projection, was also found in the proximal centralizer group than in the control group. The stem insertion time was also more prolonged compared to the control group. All these findings may indicate difficulties related to stem positioning when a proximal centralizer is used.

Similar observations were made by Goldberg et al. (1998), who concluded that in spite of a circumferential design, the proximal centralizer does not always maintain the alignment of the stem, which leads to suboptimal cement thickness between the lateral side of the prosthesis and the medullary cavity. Noble et al. (1998) have also highlighted this problem, as the lateral edge of the centralizer can become embedded in soft lateral cancellous bone, reducing cement thickness laterally. Our findings are in concordance with these remarks, indicating that a proximal centralizer may actually increase the risk of inferior alignment.

The long-term fixation of the cemented femoral stem depends on many parameters; thus, a realistic in-vitro study that could fulfill the clinical conditions would be difficult to achieve. Nevertheless, the standard true-to-life study set-up can reveal changes that occur during cementation of the femoral stem. Such studies might be beneficial before releasing a new prosthesis design onto the market.

In conclusion, we did not find any positive effects on the cementation quality during stem insertion with a custom-made proximal centralizer when high-viscosity cement was used. We found no evidence of any differences in either pressure or cement penetration between the groups. However, the risk of inferior stem positioning in the reamed medulary cavity using this centralizing device was increased.
